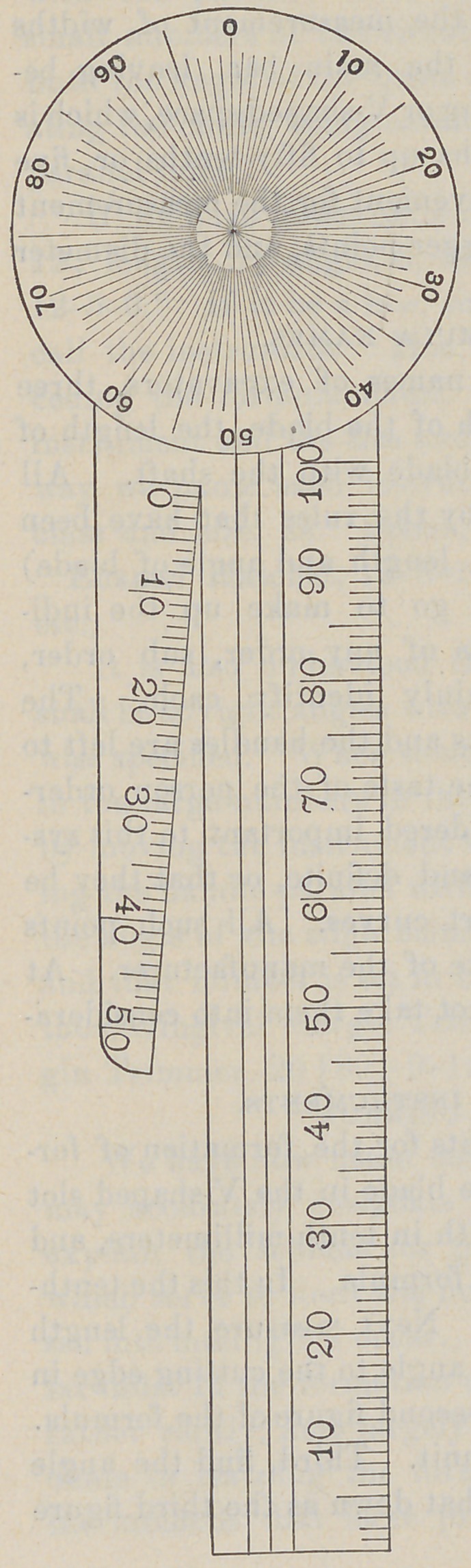# Instrument Nomenclature with Reference to Instrumentation

**Published:** 1898-04

**Authors:** G. V. Black

**Affiliations:** Chicago


### Instrument Nomenclature with Reference to Instrumentation.

BY G. V. BLACK, M.D., D.D.S., SC.D., CHICAGO.

Read before the National School of Dental Technics, Annual Meeting of 1897.

[*Continued from March No. page* 135.]

### SECOND PART.

#### FORMULA NAMES.

The names which have thus far been developed are sufficient for the designation and easy recognition of instruments belonging to any order, sub-order, class, or sub-class. They are not sufficient, however, for the recognition of the individual instruments of any one of these divisions of forms. The blade of a hatchet or hoe excavator may have an angle with its shaft varying from a slight inclination to a quarter of a circle, or even more. Any angle of blade between these may be effective for some particular operation. A similar variation occurs in the widths and in the lengths of blades. An examination of the excavators on sale in our dental depots shows that the widths of blades vary from two-tenths to fifteen-tenths millimeters. The lengths of blades vary from two to about ten millimeters. Any width or length between those mentioned may be effective in some particular operation.

Now, any of the widths may be combined with a great diversity of lengths and these again may be combined with a great diversity of angles. We readily see that in this way we arrive at a vast multitude of slight variations in these instrument forms, and any attempt to specify individual instruments, without some rules for limiting the number becomes hopeless.

I took up this matter as a subject of study a number of years ago, with the thought that these instrument forms, or a sufficient number of them, could be specified by formulæ, as is done generally with mechanics' tools: as the quarter-inch auger, half-inch chisel, etc. In this study I was at first led into a very complicated system of measurements, which I considered too complex to introduce into school work. But the need of some available system has been so constantly apparent that the subject has not been allowed to rest. Work has been renewed at intervals with each new thought obtained; and finally the idea of a strict limitation of instrument forms in breadths, lengths and angles of blades has been arrived at. The carpenter will not buy an auger or a chisel that has not been made to a definite formulæ—a definite measurement. This is true of mechanics' tools generally. They are all made to specified formulæ. It may be said that the mechanic's drills are made to definite formulæ in order that he may fit bolts made to similar definite formulæ, and that the dentist does not do this. True, but the mechanic also uses these formula in naming both his drills and his bolts that he may know them. Why should not the dentist have his instruments made to definite formulæ in order that he may know them, and designate the one fitted for a special act in excavating? Why should he have an infinite variety of forms without definiteness? No one dentist uses such a variety. Why should we not agree upon definite angles of the blades of hatchet and hoe excavators and combine with these angles definite sizes, or widths and lengths of blade? In this way we may gain a sufficient number of forms of cutting instruments and rule out all others. And then the thought has also come to me of arranging these in definite sets in which the formula names shall run on definite gradations for all of the instruments of each set, and in this way so construct them that they will be easily learned and remembered by students.

A strict study of the subject from this standpoint develops the fact that we do not need more than three, or at most four angles. Now with each of these three or four angles we will combine one long blade of definite width, one medium length of definite width, and one short blade of definite width, stipulating that the lengths and widths shall be the same in each angle. This makes a set of hatchets—if three angles be used—of nine instruments, and a set of hoes of nine instruments—or eighteen instruments in all. These we may name the set of ordinaries. (See list of formulæ No. 4.) With this limitation of widths and lengths and angles of blades, and the regular order in which they occur, the difficulty of learning to know them by formulæ is reduced to a minimum. Indeed it is found in actual practice that the forms are known by sight as quickly as this simple list of formulæ is learned.

I have chosen and had made some sets of instruments upon this idea, and find from actual use that three angles is quite enough for my personal use. It is necessary only to add a list of spoons, enamel instruments, and a few long blades for reaching into deep cavities, to make the set complete. A list of special forms for special uses, the formulæ for which are constructed upon a similar plan.

It will be seen now, I think, that the infinite variety of widths, lengths and angles of blades without definiteness or restriction of any kind, except the fancy of those ordering instruments, is responsible for the chaotic condition of the forms of cutting instruments. It is my belief that for school work a strict limitation of instrument forms to those that may be accurately designated is desirable.

SELECTION OF SYSTEM OF MEASUREMENT.

If we have decided that a system of formulæ based upon measurements of widths, lengths and angles of blades is desirable, the next point will be to agree upon the particular system of measurement to be adopted.

For the measurement of widths and lengths we have the English inch and the French millimeter. Of these I should choose the French system for two reasons. *First*, from the present indications, it seems that it will in time become the only system employed in scientific work. *Second*, the length of the unit seems much more convenient for the work ; particularly is this the case if we use the tenth of the millimeter for all measurements of breadths and the millimeter for all measurements of lengths of blades. This seems to be so evident that I have adopted this pending discussion.

The adoption of a system of graduation of the circle for the measurement of angles is a graver problem. The astronomical circle with its graduation of 360 degrees is far in excess of our needs, and becomes cumbersome because of the minuteness of its sub-divisions. On the other hand, it is the division of the circle most used and best known. The mariner's compass with its division of the circle into thirty-two points seems insufficient. The division of the circle into 100, the centigrade circle, seems very much better suited to our needs. In this, 25 centigrades is a quarter of a circle, and equal to 90 degrees of the astronomical circle. The quarter circle is about all that we use, and the graduations of this are much more quickly caught and appreciated than in the larger number of divisions. I shall use this pending further discussion.

THE GAUGE.

With the view of making the preparation for this work as nearly perfect as possible, I have had a gauge made in steel for instrument measurement. It consists of a circular head graduated in hundredths, and an attached bar ruled in parallel lines for the measurement of angles. The bar is also graduated in millimeters for the measurement of lengths. For the measurement of widths a supplemental bar extends beside the main bar, leaving between the two bars a gradual widening or V-shaped space, which is graduated in tenth-millimeter widths up to fifty-tenths or five millimeters. This is found very convenient for the measurement of widths of blades, the sizes of plugger points, and the diameter of burs.

### FORMATION OF FORMULA NAMES.

For the formation of formula names of excavators, three points are considered, viz: the width of the blade, the length of the blade, and the angle of the blade with the shaft. All other points are left to be guided by the rules that have been given in Part First. These (width, length and angle of blade) are very exactly the points that go to make up the individuality of the several instruments of any order, sub order, class or sub-class, and will certainly identify each. The particular conformation of the shanks and the handles are left to the individual manufacturer, or to the taste of the person ordering instruments. Neither is it considered important to this system that the angles be made sharp and definite, or that they be made in the form of moderately short curves. All such points in construction can be left to the taste of the manufacturer. At least the system now proposed does not take them into consideration.

### THE MEASUREMENT OF INSTRUMENTS.

In the measurement of instruments for the formation of formula names, first try the width of the blade in the V-shaped slot of the gauge, which will give the width in tenth-millimeters, and set this down as the first figure of the formula. In this the tenth-millimeter is to be used as the unit. Next measure the length of the blade from the center of the angle to the cutting edge in millimeters and set that down as the second figure of the formula. In this the millimeter is used as the unit. Third, find the angle of the blade with the shaft and set that down as the third figure of the formula. In making this last measurement, lay the handle of the instrument on the main shaft of the gauge, parallel with the parallel lines, and with the point turned toward the small numbers of the circular head. Now move the instrument until the angle of the blade coincides with one of the lines graduating the circle, being careful to keep the handle parallel with the parallel lines.

If we have measured a hatchet and the numbers give—width, 12; length, 5; angle, 6, the formula name will read "Hatchet, 12-5-6." If it be a hoe, the formula will be the same and we call the instrument "Hoe, 12-5-6," the class name always preceding the formula name. This distinguishes both the kind of instrument and the size and angle of the blade of each. In this way we name each instrument of the set, no matter what its class and size, as "Spoon, 20-9-12," or "Spoon, 15-8-12," or "Enamel Hatchet, 15-8-12," or "Enamel Hatchet, 10-6-12," etc.

It is also understood that the edge of cutting instruments shall be at right angles with the length of the blade, unless otherwise specified. When some other angle is desired, it is measured in the large numbers in the last quarter of the graduated circle by moving the instrument without turning it over, and still keeping the handle parallel with the parallel lines of the gauge until the angle of the edge coincides with one of the centigrade lines, and that number is set in brackets following the width number, thus, Gingival Margin Trimmer, 20 [95]-9-12 or Gingival Margin Trimmer, 20 [80]-9-12.

### FORMING INSTRUMENT LISTS.

We have now made out rules of nomenclature by which we may accurately designate individual instruments. I will now explain the scheme for grouping instruments in formula lists which serve to limit the number of forms and to bring those chosen into intelligible order. The appreciation of the value of regular order in the formation of instrument sets has been arrived at rather slowly, and largely from studying the difficulties of students in learning the forms of their instrument points. With the methods that have prevailed few persons learn to think in their instrument forms. They have to search for the proper instrument instead of reading it in the case before them. It is that we may be able to teach pupils to think in their instrument forms that we strive to construct graded sets in formula nomenclature; and these should be placed on such lines of gradation, or be so grouped, that the mind easily follows from one to another throughout the set.

It is not difficult to do this with any of the forms of excavators, but some of them are more easily arranged than others. The ordinary hatchets and hoes present the greatest variations of size and angle of blades, but fortunately are the most easily graded into sets. Carpenters' augers are made in gradations of sizes of 1-32d inch, making the most perfect set. Another set is made on gradations of 1-16th inch, this set containing but half the number of the first. Still another set is made on gradations of 1-8th inch, containing but one-fourth the original number. Yet each of these sets is complete upon its individual lines, and each of the smaller sets is contained in the larger.

For the ordinary hatchet and hoe excavators we may readily do a similar thing by first constructing a list of formulæ on regular gradations that will cover the useful sizes and angles of blades, and then cut out all of certain dimensions or angles in the formation of shorter lists. This is not so readily done in spoons, enamel hatchets and some other forms, for the reason that in these we do not require so many instruments of a given class. These also require different formula names, for the reason that the blades are of different dimensions from those of the hatchets and hoes. They must therefore be placed in a different formula list in which we can group together such instruments as agree in dimensions of blade. If necessary, we may make several formula lists. At present I will propose three divisions, naming each, as follows:

*Ordinaries* are the common forms of hatchets and hoes, many of which are found in every operating case.

*Specials* are those instruments designed for special acts in excavating, such as spoons, enamel hatchets, chisels, etc.

*Side Instruments.*—These are selections for some particular purpose, only one or two of which are wanted in the instrument set, and which it is not desirable to include in a regular formula list.

ORDINARIES.

After a long and careful study of the dimensions, proportions and angles of blades of the hoe and hatchet excavators used by dentists and generally on sale in dental depots, I am of the opinion that nearly or quite every dentist will find in the following formula list about everything he will want:

SET OF ORDINARIES, NO. 1.

14-6-6, 12, 18 and 23.
12–5
10–4
8–3
6–2
4–1
forty-eight instruments.

Formula lists for ordinaries will be given in this form. The first figure gives the width of blade; the second the length of blade; the third the angle of the blade with the shaft; and the additional angles used are given in the first line only, divided by commas.

Each of the dimensions of blade is to be made in each of the angles given both in hatchets and hoes. The list is to be read: Hatchet 14–6–6, hatchet 14-6–12, hatchet 14-6–18, hatchet 14–6–23; or hoe 14–6–6, etc., for the first line; and hatchet 12–5–6, hatchet 12–5–12, hatchet 12–5-18, hatchet 12–5–23; or hoe 12–5–6, etc., for the second line. This is continued in the same way for each of the dimensions of blade. *The formula of each instrument is stamped upon its handle as a convenience to the student in learning his instrument points.*

According to the rules for contra-angling given in Part First, hatchet and hoe 14–6-12 would be binangle contra-angles. Also hatchets and hoes 14–6–18 and 23,
12–5
10–4 would be triple angle contra-angles.

There are in the set twenty-four hatchets and twenty-four hoes, or forty-eight in all, and if generally adopted as the full list of ordinaries, would, I think, be found satisfactory.

In making shorter lists I would cut out all of certain dimensions of blade, or of certain angles, preserving the regular order of formula names for those retained. As the least desirable I would first remove all of dimensions 14–6 and 4–1, thus:

SET OF ORDINARIES NO. 2.

12–5–6, 12, 18 and 23.
10–4
8–3
6–2
thirty-two instruments.

This set is a most beautiful gradation of the ordinary forms of excavators, and really embraces about all that any dentist would want in his case. But these are probably a greater num ber than most persons would desire.

For the next set I would remove all of the dimensions 10–4, thus:

SET OF ORDINARIES NO. 3.

12–5–6, 12, 18 and 23.
8–3
6–2
twenty-four instruments.

This is also a very effective instrument set, but if there are still too many I should remove all of the angle 18 centigrades, thus:

SET OF ORDINARIES NO. 4.

12–5–6, 12 and 23.
8–3
6–2
eighteen instruments.

This I regard as an especially desirable list for school work. It is the list I have used most except that I have used the dimensions 5–2 instead of 6–2, but in the future will use the 6–2.

Now, for a still shorter list, and the shortest that I could recommend as reasonably efficient, I would retain but two dimensions:

SET OF ORDINARIES NO. 5.

10–4–6, 12 and 23.
6–2
twelve instruments.

This is a list of six hatchets and six hoes excellently graded to the requirements of the student—indeed I do not know how we could better select this number of instruments.

In the instrument sets given we have five, differing widely in numbers, but in each the formulæ are complete on the lines laid out and every instrument is a good one. The smaller sets are all contained in the largest, and are so arranged as to give manufacturers the least trouble in supplying classes. If manufacturers will make up List No. 1, or even List No. 2, and make these their stock instruments in ordinaries, there are few wants in this line that will not be supplied by them. From them any school that may desire to introduce the formula plan of nomenclature in teaching will be able to choose a satisfactory list. Within a few years this may become the plan of the dental profession, and the manufacturers will be relieved from the loads of dead instrument stock they are now compelled to carry. That other instruments in this line will be demanded goes without saying, but they will be fewer in number as discussion of plans and methods under conditions of greater accuracy of understanding proceeds.

SPECIALS.

In the list of specials I will give such only as I have defined in Part First. These seem to me from my personal study and use of cutting instruments to be best suited to our present methods of preparing cavities. I will first give what I regard as a complete list, and afterward cut it down to smaller numbers, removing such instruments as can be spared with the least detriment to effective school work. It is to be understood that each full instrument set is to contain a list of ordinaries and a list of specials. The list of specials will contain numbers of classes instead of a great variety of sizes and angles of two classes, as is the case with the ordinaries. We do not require many sizes and angles of blade in any one class of specials. After a careful study of them it is found that most of them may be arranged upon practically the same formula numbers. There are a few, as the straight chisels and the cleoids, which will not require the full formula terms to sufficiently designate them. Three widths of blade seem to me to be the most that will be necessary and nearly all may be of the angle 12 centigrades, a few only requiring the angle 6 centigrades. The length of blade may be on the same lines in all but the discoids, the length and breadth of which are necessarily the same.

LIST OF SPECIALS NO. 1.

| | |
|---|---|
| Enamel hatchets . . . . . . | 20-9-12 Pr. R. & L. bevels |
| Enamel hatchets . . . . | 15-8-12 Pr. R. & L. bevels |
| Enamel hatchets . . . . . . | 10-6-12 Pr. R. & L. bevels |
| Spoons . . . . . . . . | 20-9-12 Pr. R. & L. curved |
| Spoons . . . . . . . . . . | 15-8-12 Pr. R. & L. curved |
| Spoons . . . . . . . . | 10-6-12 Pr. R. & L. curved |
| Spoons . . . . . . . . . . | 20-9-6 Pr. R. & L. curved |
| Spoons . . . . . . . . | 15-8-6 Pr. R. & L. curved |
| Spoons . . . . . . . . . . | 10-6-6 Pr. R. & L. curved |
| Gingival margin trimmers . | 20 (95)-9-12 Pr. R. & L. curved |
| Gingival margin trimmers . . | 20 (80)-9-12 Pr. R. & L. curved |
| Gingival margin trimmers . | 15 (95)-8-12 Pr. R. & L. curved |
| Gingival margin trimmers . . | 15 (80)-8-12 Pr. R. & L. curved |
| Binangle chisel . . . . . . | 20-9-6. One instrument |
| Binangle chisel . . . . . . | 15-8-6. One instrument |
| Binangle chisel . . . . . . | 10-6-6. One instrument |
| Straight chisel . . . . . . | 20. One instrument |
| Straight chisel . . . . . . | 15. One instrument |
| Straight chisel . . . . . . | 10. One instrument |
| Discoid . . . . . . . . | 20-2-12 |
| Discoid . . . . . . . . . . | 15-1½-12 |
| Discoid . . . . . . . . | 10-1-12 |
| Cleoid . . . . . . . . . . | 20 |
| Cleoid . . . . . . . . . . | 15 |
| Cleoid . . . . . . . . . . | 10—thirty-eight instruments. |

This gives a list of thirty-eight special instruments. Several other forms might be added, but to me they seem unnecessary. They can be added, however, upon the same plan of formulæ used in this list, or if necessary still another formula list may be arranged. This list will give rise to more difference of opinion than the list of ordinaries, for the reason that they are designed for special uses in excavating, and persons who excavate cavities differently are likely to want different special forms. Such differences, however, have no reference to the formula plan of nomenclature, as other forms can as readily be brought into this system.

In this list of specials each instrument is designed for the performance of a special act in excavating. The enamel hatchets are designed for chipping enamel by hand pressure in opening cavities in the bicuspids and molars. They are beveled rights and lefts and are somewhat distinctive in form and use. When the manner of handling them and their adaptation to place of use has been learned, they are unusually effective instruments. Indeed, besides their use in chipping enamel, they become the principal instruments for cutting out and forming both mesial and distal cavities in the bicuspids and molars, both upper and lower. Their angle of blade and form of edge is such that they naturally cut these cavities into proper form. And when properly supplemented by burs, they are very effective in extending these cavities for the prevention of the recurrence of decay at the gingival margin, or at the bucco-gingival and linguo-gingival angles.

The spoons are for the removal of carious or softened material in any position, but more especially in the large cavities in the bicuspids and molars, also for uncovering exposed pulps the broader blades are invaluable. Of these spoons the pairs in 12 centigrades angle seem to be preferred, though the 6 centigrades angle are the instruments heretofore generally in the market.

The gingival margin trimmers, two pairs of which are of one size, and another two pairs of another size, are for the one purpose of smoothing and beveling the marginal angle of the gingival wall in proximate cavities in the bicuspids and molars. For this purpose they have the cutting edge ground to a definite angle with the shaft. This is made 80 centigrades in the one pair, which fits them for mesial cavities, and 95 centigrades in the other pair, which fits them for distal cavities. The smaller pairs serve this purpose in places too narrow for the entrance of the 20 tenths width of the larger. These are the only instruments in the list that have cutting edges other than at right angles with the length of the blade.

Of chisels I have placed six on the list. Three of them are straight, and the width of blade only is given in the formula name, as chisel 20, or chisel 10. All have cutting edges at right angles with the shaft. Those designated as "binangle chisels" have the full formula name with an angle of 6 centigrades. They are so contra-angled as to bring the working edge in the line of the shaft. The six form a very effective set for chipping enamel in the opening of cavities, and in trimming the walls to form. The angles of the binangle forms adapt them admirably to the trimming of buccal walls in molars and bicuspids in places where a slight angle of blade is necessary to reach the best position for cutting.

The discoids perform much the same office as spoons, and are available in positions of easy access. When direct access can be had, they are to be preferred.

The cleoids are available for almost any purpose demanding a pointed instrument. I use them much in opening pulp-chambers in upper bicuspids, and in beveling lingual enamel margins in incisors, also frequently in following out fissures in the molars.

In forming sets of these of fewer numbers I would first cut out the list of spoons in 6 centigrades angle; second, the list of cleoids, and third, the discoids; fourth, the gingival margin trimmers 15(95)-8-12 and 15(80)-8-12, leaving the list stand thus:

SET OF SPECIALS NO. 2.

| | | |
|---|---|---|
| Enamel hatchets . . . . | 20-9-12 | Pr. R. & L. bevels |
| Enamel hatchets . . . | 15-8-12 | Pr. R. & L. bevels |
| Enamel hatchets . . . . | 10-6-12 | Pr. R. & L. bevels |
| Spoons . . . . . . . . | 20-9-12 | Pr. R. & L. curved |
| Spoons . . . . . . . . | 15-8-12 | Pr. R. & L. curved |
| Spoons . . . . . . . | 10-6-12 | Pr. R. & L. curved |
| Gingival margin trimmers . | 20 (95)-9-12 | Pr. R. & L. |
| Gingival margin trimmers . | 20 (80)-9-12 | Pr. R. & L. |

| | |
|---|---|
| Binangle chisel . . , . | 20-9-6 |
| Binangle chisel . . . . | 15-8-6 |
| Binangle chisel . . . . | 10-6-6 |
| Straight chisel . . . . . | 20 |
| Straight chisel . . . . | 15 |
| Straight chisel . . . . . | 10—twenty-two instruments. |

For a still shorter list, and the shortest list of specials that I could recommend, I would cut out from set No. 2 all of the dimensions 10-6, thus:

SET OF SPECIALS NO. 3.

| | |
|---|---|
| Enamel hatchets . . . . | 20-9-12 Pr. R. & L. bevels |
| Enamel hatchets . . . . | 15-8-12 Pr. R. & L. bevels |
| Spoons . . . . . . . . | 20-9-12 Pr. R. & L. |
| Spoons . . . . . . . | 15-8-12 Pr. R. & L. |
| Gingival margin trimmers . | 20 (95)-9-12 Pr. R. & L. |
| Gingival margin trimmers . | 20 (80)-9-12 Pr. R. & L. |
| Binangle chisel . . . . | 20-9-6 |
| Binangle chisel . . . . . | 15-8-6 |
| Straight chisel . . . . | 20 |
| Straight chisel . . . . . | 15—sixteen instruments. |

This list is really quite effective, though one who has become accustomed to the smaller sizes will miss them.

Of these lists No. 2 of the specials, combined with No. 4 of the ordinaries, makes an excellent set for school work. It contains thirty-four instruments, every one of which will come into active use in the ordinary infirmary practice.

Also set of specials No. 3, combined with set of ordinaries No. 5, makes a well-chosen short set of twenty-eight instruments that is quite effective for school work, though some very desirable instruments are missing.

These lists are extremely simple in their formula nomenclature and are easily learned by pupils. Of course, other combinations of these lists may be made at will. Yet it is important that the direct relation of the formula names be carefully maintained in any lists made up for school use.

SIDE INSTRUMENTS.

Side instruments should be made to definite formulæ, that they may receive definite names. For instance, in breaking up the list of specials for the formation of smaller lists, discoid 20-2-12 may be retained as a side instrument, or one of the cleoids may be retained. I like to have in the instrument list as side instruments hatchets 5-3-28 and 3-2-28 for cutting retention grooves in the incisal angle of incisor cavities. It will be noticed that the formulæ of these latter do not follow the lines of the list given. The number of such instruments added to working sets in schools should be limited to a very few favorite forms for some special use. Any considerable number of them will certainly cause confusion in the minds of students, and interfere with the easy mastery of the list as a whole.

Other formula lists may be added when desired. This year I have used an additional list of long, slender blades expressed thus:

Hatchets and hoes—12-8-12 and 23.
8-6.

Of these the blades in 12 centigrades angle are most excellent instruments for deep cavity work, and yet my experience thus far in teaching leads me to the conclusion that the introduction of this third formula list is undesirable. In other words, instruments in the other two lists so nearly take the place of these that it seems undesirable to burden the students with this additional list.

There is really no limit to the number of lists that might be formed by this method, and if I have now made this clear I have finished my task in this direction. But the more important consideration is the limiting of the instrument forms to definite lines, easily followed by the student and readily supplied by the manufacturer.

It must be distinctly understood that in ordering instruments by the formula plan, the class name of each instrument must be given with its formula—as Hatchet 12-5-6, or Spoons 20-9-12.

It seems very desirable that some rule be established as to which instrument shall be called the right or the left in the instrument pairs. I will suggest that this be based on convenience of use in the right hand. That blade which, when held as a pen with the point downward, has the convex side of the blade to the right is called the right-hand instrument; and the blade which has the convex side of the blade to the left is the left-hand instrument. In beveled rights and lefts the beveled side corresponds to the convex side of curved blades.

TEACHING INSTRUMENTS AND INSTRUMENTATION.

When the time came for opening school this year, I felt that I could not begin without putting the plan for formula names to trial. The teaching of the mechanical forms, the adaptation of forms to the ends to be accomplished and plans of instrumentation were begun in Northwestern University Dental School this year under extreme disadvantage. It was really impossible that it should be otherwise in the beginning. It has come upon a class of three hundred and fifty pupils—juniors and seniors—after they have accomplished a part of their course by other methods, and with instruments of different forms. To make matters worse, on account of the slowness of manufacturers, together with the extraordinary demand for the particular instrument set used, only a portion of the pupils could be promptly supplied. This has been a great drawback to effective work. Yet the experience gained thus far has been a most valuable study of the effectiveness of the method and of the plans to be employed in teaching. Most pupils who obtained their instruments in time learned to read their points readily and have made rapid progress in instrumentation.

The proper place to begin this teaching is in the operative technic class; and for this purpose the pupil should be required to obtain his cutting instruments in his freshman year. One of the first and most important steps is to give the pupil a good working knowledge of the value of the millimeter, of tenths of a millimeter, and of centigrade angles. He should attain this in such degree that he will be able to cut bits of paper, or of some soft metal, five, ten or fifteen-tenth millimeters wide, or five or ten millimeters long with reasonable accuracy without the use of the gauge; and to form any given angle. In this study he must first work with the gauge or with the printed form. A very excellent instrument for this study is the Boley gauge, an instrument that is specially well adapted to measuring teeth, and many other things in school work and in the dental office. As this is being accomplished the instrument forms are presented one by one, as hatchets, spoons, hoes, etc., and the mechanical features of each, the nomenclature of its different parts, and the relation of the instruments to each other explained. The capabilities of each form will be familiarized by exercise in their use in carving in bone, and forming cavities in teeth. In doing this, correct instrument grasps, and finger and thumb rests, will be taught. The pupil is then presented with the various sizes of each form and learns to distinguish them and to use their formula names.

In this way the pupil becomes fitted to enter the junior year in which this teaching begins to be put into actual practice in the mouth. Now a review of the instrument forms, their nomenclature, and the uses of each, is made in connection with the teaching of the preparation of cavities. In this the lecturer and the demonstrator at the chair become able to direct the student effectively, so that his use of instruments is begun correctly, and comparatively rapid progress made on right lines. This much-neglected branch of operative dentistry, instrumentation, can now be taught effectively.

Cavity preparation, in my conception of it, should proceed in a definite order, step by step, which a student should be taught to observe strictly, to carry out with certain instruments, and with fairly definite methods of instrumentation. It is only when he is able to accomplish this work upon a definite system that he should be regarded as able himself to form his lines of procedure in such a manner as will lead him to that high degree of skill in the future which we desire that our pupils should attain.

## Figures and Tables

**Figure f1:**